# Defining key interventions for rectal cancer surgery: a literature review and expert panel consensus

**DOI:** 10.1007/s00384-025-05028-z

**Published:** 2025-12-02

**Authors:** Cédric Schraepen, Gabriele Bislenghi, Kris Vanhaecht, André D’Hoore, Albert Wolthuis, Ellen Coeckelberghs

**Affiliations:** 1 Department of Abdominal Surgery, University Hospitals Leuven, Leuven, Belgium; 2 Department of Public Health and Primary Care, Leuven Institute for Healthcare Policy, KU Leuven– University of Leuven, Leuven, Belgium; 3 Department of Quality Management, University Hospitals Leuven, Leuven, Belgium

**Keywords:** Rectal cancer surgery, Key interventions, Abdominal surgery, Quality of care

## Abstract

**Background:**

Despite advances in minimally invasive techniques and widespread adoption of Enhanced Recovery Programs (ERPs), rectal cancer surgery continues to pose significant challenges due to anatomical limitations, risks of complications, and the potential impact on bowel, urinary, and sexual function. These complexities underline the need for clearly defined, evidence-based key interventions to assess and ensure consistent, high-quality care across institutions. The aim of this study is to identify and summarize the evidence-based key interventions relevant to rectal cancer surgery.

**Methods:**

A focused PubMed/MEDLINE search was performed to identify key interventions in care pathways for rectal cancer surgery. The list of key interventions extracted from the literature was presented to an expert panel who evaluated their importance and relevance in a one-round Delphi process.

**Results:**

In total, 293 papers were screened on title and abstract for relevant information. Twelve papers were retained to identify the initial set of key interventions (n = 56). The list was narrowed to 39 by excluding duplicates and outdated key interventions. This list was commented on during a 1-round Delphi, and consensus was reached for 37 key interventions regarding surgical rectal cancer treatment.

**Conclusion:**

We propose this list of 37 key interventions as a contemporary framework for assessing rectal cancer surgery.

## Introduction

Despite minimally invasive surgery (MIS) and Enhanced Recovery Programs (ERP), rectal cancer surgery continues to carry a substantial risk of postoperative morbidity and mortality [1–4]. In addition to patient-related factors, organizational and process elements—such as high surgeon and hospital volumes and consistent use of a multidisciplinary team (MDT)—are associated with improved oncological and functional outcomes [5, 6]. However, translating evidence-based recommendations into routine, reproducible practice remains challenging.

Care pathways are an established method to embed complex perioperative interventions and to reduce unwarranted variation. Multiple studies have shown that well-designed pathways improve surgical outcomes and resource utilization [7–10]. For example, Howell et al. demonstrated that pathway-driven interventions can reduce adverse events in surgery by standardizing critical perioperative steps [9], and Seys et al. highlighted their value for reliable implementation across institutions [11].

Several international frameworks now define contemporary standards for high-quality rectal cancer care. These include the National Accreditation Program for Rectal Cancer (NAPRC) of the American College of Surgeons (ACS) and the Optimizing the Surgical Treatment of Rectal Cancer (OSTRiCh) consortium, the Enhanced Recovery After Surgery (ERAS®) Society’s guidelines, and the American Society of Colon and Rectal Surgeons (ASCRS) clinical practice guidelines. Together, NAPRC, ERAS®, and ASCRS provide complementary, evidence-based benchmarks for perioperative management and quality assurance in rectal cancer surgery [1, 6, 12]. In contrast, our approach focuses on patient-level key interventions—specific clinical actions within the preoperative, intraoperative, and postoperative phases (including 90-day outcomes) that can be incorporated into local care pathways and form the basis for future quality indicators. By identifying and implementing patient-centered key interventions, we aim to complement existing system-level interventions and contribute to their improvement from the base. This complementary perspective emphasizes the importance of aligning institutional standards with the individual patient journey.

The objective of this study was to identify and prioritize such essential clinical interventions for elective rectal cancer surgery through a structured, guideline-based development process as described by Kötter et al. [13], combined with a national expert consensus. While not all these key interventions are directly measurable as quality indicators, they form a clinically validated basis for future quality monitoring and improvement efforts.

### Key message

This consensus-based framework provides clinicians and institutions with a practical, reproducible set of 37 patient-level interventions for rectal cancer surgery. It helps integrate evidence-based steps into local care pathways, supports consistent 90-day outcome monitoring, and offers a foundation for future quality indicators and care pathways.

## Methods

**Fig. 1:**
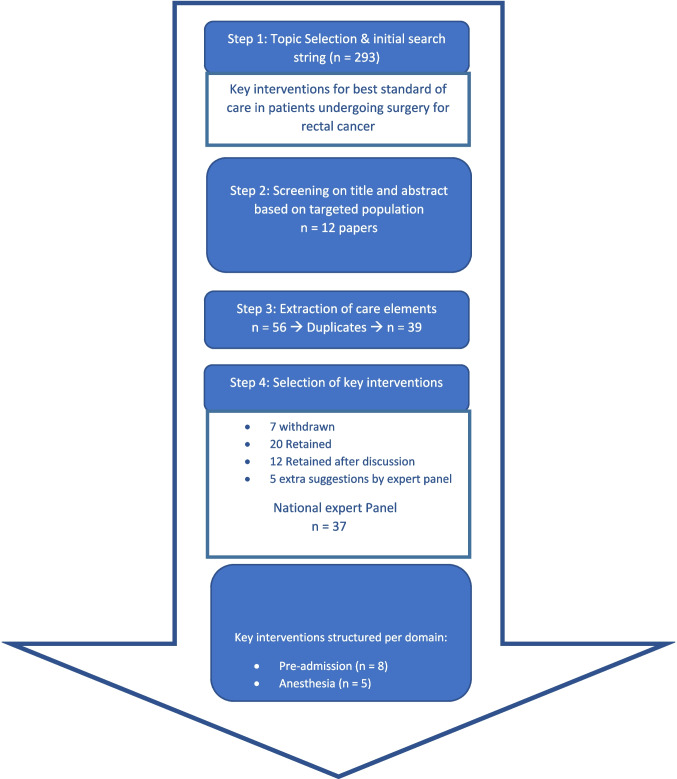
Schematic overview of literature review

A systematic and transparent approach is necessary to develop key interventions relevant to the quality of care in rectal cancer surgery. These key interventions need to be scientifically acceptable, feasible, clinically relevant, usable and allow for discrimination [14, 15]. We used the stepwise approach for a guideline-based development of key interventions as proposed by Kotter et al. [13]. They recommend six sequential steps: Topic selection, guideline selection, extraction of recommendations, quality-indicator selection, practice test and finally implementation. The present project reports on the first four steps, as testing and implementation will follow in future work. A visual summary of the process and article selection is provided in Fig. 1.

In the first step, a topic was selected and criteria for narrowing it down were identified by the research team and a group of faculty rectal cancer surgeons (AW, ADH, GB) in a tertiary referral center. In the second step, a selection of guidelines from peer-reviewed literature was made. Third, key interventions from the guidelines and literature were extracted. In the fourth step, we conducted a single-round Delphi consensus among 25 visceral surgeons involved in colorectal surgery, since colorectal surgery is not a distinct subspecialty in Belgium. The panel included surgeons from four Belgian university hospitals as well as peripheral hospitals, providing a balanced mix of academic and non-academic expertise. All invitees completed the survey (response rate 100%). All participants were senior staff colorectal surgeons, most of them heads of their colorectal units. Based on professional profiles, each had at least ~ 10 years of independent practice. Exact age and individual operative volumes were not collected. Each candidate intervention was rated on a 10-point Likert scale (1 = not relevant, 10 = indispensable). Consensus was predefined as ≥ 75% of experts scoring an item ≥ 8. Items scoring < 4 were excluded; items scoring 4–7 were discussed during a live video panel and included if consensus was reached. Panelists could also propose new interventions. No conflicts of interest were declared.

## Results

### Step 1: Topic selection

The topic, “Key interventions for best standard of care in patients undergoing rectal cancer surgery” was defined by the research team (CS, EC) together with three faculty colorectal surgeons in a tertiary referral center (AW, ADH, GB). The target population comprised adults scheduled for elective rectal cancer surgery, including total mesorectal excision (TME) and abdominoperineal resection (APR). The following research topics were excluded: patients with metastatic disease, patients with synchronous (colorectal) cancer and patients with underlying diseases like Familial Adenomatous Polyposis (FAP) or inflammatory bowel disease (IBD) specifically. While the focus was on identifying clinically relevant perioperative interventions, the potential impact on resource use and patient quality of life was considered when reviewing the literature and formulating candidate interventions.

### Step 2: Literature review and selection of guidelines

To identify candidate interventions, the principal investigators (EC, CS) independently conducted an extensive literature review to determine key interventions for the best standard of care for all patients undergoing rectal cancer surgery. The primary database was PubMed/MEDLINE, searched on October 14th 2024 with the following MESH terms: ‘"Patient Reported Outcome Measures"[Mesh] OR ((("Guideline Adherence"[Mesh]) OR "Guideline" [Publication Type]) OR "Quality Indicators, Health Care"[Mesh]) AND "Rectal Neoplasms"[Mesh]’’. No duplicates or automation-based exclusions were identified. After title and abstract screening, 281 records from the initial 293 were excluded due to not focusing on rectal cancer surgery, lacking explicit clinical recommendations, or being non-English. Of the remaining 34 reports sought for retrieval, one could not be retrieved. Full-text assessment of 34 reports led to the exclusion of 21 other articles (no clinical recommendations, not within the scope of step 1). Ultimately, 12 key publications were included, from which the first set of key interventions was extracted (Fig. 1, Annex 1).

### Step 3: Extraction of key interventions

A first set of key interventions (n = 56) was categorized into 5 sub-categories that cover the perioperative setting in rectal cancer surgery: pre-admission, anesthesia, surgery, postoperative course and clinical outcome. After elimination of duplicates, the list was reduced to 39 specific key interventions.

### Step 4: Final selection of the relevant key interventions

From the 39 candidate interventions presented to the panel, three were excluded for low relevance (< 4). Twenty were included immediately (≥ 8), and twelve were retained after discussion of intermediate scores (4–7). The panel also proposed five new interventions, resulting in a final list of 37 key interventions. Highest consensus was reached for multidisciplinary team consultation (97%), preoperative patient counseling (89%), postoperative tumor board (94%), anastomotic leakage (89%), re-intervention (90%), and 90-day mortality (93%) (Tables 1 and 2). An illustrative mapping of interventions to possible quality indicators, evidence levels, and data sources is provided in Annex 3 to facilitate future indicator development.

**Table 1:** Overview of included key interventions with median and mean-score by expert panel (*n* = 37)

Key intervention	Median	Mean
**Pre-admission**		
- Preoperative MDT - Patient-education and counseling - Stoma education and positioning - Optimization of general condition (smoking stop, risk assessment, probiotics) - Screening nutrition status - Oral nutrition supplements - Screening and treatment of anemia - Preoperative MRI**	10 9 9 8 8 8 9	9.7 8.9 8.7 7.7 7.6 7.8 8.4
**Anesthesia﻿**		
- Intravenous antibiotic prophylaxis - Intraoperative fluid balance - PONV Screening (Apfel score) * - PONV prophylaxis* - Avoiding sedative medications*	9 8 7 7 7	8.7 7.7 6.8 7.2 6.9
**Surgery (intraoperative)**		
- Bowel preparation (with or without antibiotics) - Disinfection of the skin* - Minimally invasive surgery - Type of approach: open/laparoscopic/robotic - Type of reconstruction* - Splenic flexure mobilization* - Visual check anastomosis* - Under water leakage test* - Extraction site location* - Use of Indocyanine green (ICG)* - Selective drain placement* - Stoma placement* (temporary or terminal) - High vs low tie mesenteric vein/artery**	8.5 7 9 8 6 7 6 7 7 7 5 7	8.2 6.7 8.7 7.9 5.5 6.9 5.2 7.7 7.2 6.5 4.8 7.1
**Postoperative course**		
- Multimodal pain assessment - Placement and removal of bladder catheter - Postoperative mobilization - Postoperative nutrition - Anastomotic leakage	8 8 9 8 9	8.2 7.3 8.8 7.6 8.9
**Clinical outcome**		
- Re-intervention - 90-day readmission/mortality - Postoperative MDT - Quality of TME Specimen** - Pathology features** - Time to stoma closure**	9 10 10	9 9.3 9.4

*Accepted after expert panel discussion

**Added by expert panel

**Table 2:** Overview of excluded key interventions with median and mean-score by expert panel (*n* = 7)

Key intervention	Median	Mean
**Pre-admission**		
- Prehabilitation* defined as a multidisciplinary intervention aimed at optimizing the physical, nutritional, and psychological status of a patient prior to surgery	6.5	6.9
**Anesthesia**		
- Perioperative fluid balance* - Sober before surgery (6 h for solid, 2 h for liquids) *	6.5 7	6.8 6.8
**Surgery (intraoperative)**		
- Nasogastric tube placement - Trans anastomotic catheter placement - Reinforcement of anastomosis by additional sutures	2 2 2	3.1 2.8 3.0
**Postoperative course**		
- Multimodal prevention of ileus	7	7.1

*Withdrawn after expert panel discussion

## Discussion

This study defined a contemporary set of 37 key interventions for rectal cancer surgery through a literature review and a single-round national Delphi process. The resulting framework provides a reproducible basis for integrating evidence-based steps into rectal cancer care pathways and for developing future quality indicators.

### Alignment with international standards

High consensus on pre- and postoperative multidisciplinary team (MDT) consultation (97% and 94%, respectively) underscores the central role of coordinated decision-making in rectal cancer care. MDTs comprising surgeons, radiologists, pathologists, and (radiation) oncologists enable comprehensive treatment planning and have been associated with reduced recurrence and improved survival. These findings are consistent with the standards of the NAPRC, which mandates multidisciplinary evaluation and systematic quality monitoring [6, 16]. Surgical and postoperative outcomes, such as anastomotic leakage (89%), re-intervention (90%), and 90-day mortality (93%), were given high scores by the expert panel. Additionally, postoperative complications often necessitate re-interventions, leading to prolonged hospital stays and negatively affecting long-term survival. Therefore, vigilant monitoring of these indicators is essential to promptly identify and address complications, ultimately enhancing patient outcomes [17]. These findings are consistent with ERAS ® and ASCRS guidelines emphasizing perioperative optimization and robust 90-day outcome monitoring [1, 12].

### Excluded or reclassified items

In our study, several key interventions were excluded due to limited or inconsistent evidence regarding their effectiveness. Prehabilitation was not retained, largely because current programs differ widely in duration, intensity, and multimodal components, making standardization and outcome evaluation difficult. Nevertheless, emerging trials show potential benefit, indicating a need for further high-quality studies to clarify its role [18, 19]. Similarly, high versus low tie of the inferior mesenteric artery/vein was ultimately retained, although current literature shows no clear survival benefit, likely serving more as a data-collection item or technical variable than as a true key intervention [20]. Perioperative fluid balance and soberness were excluded by the experts due to varying recommendations and a lack of consensus on optimal management strategies in the perioperative setting [21]. The practice of reinforcing anastomosis with additional sutures was also excluded, as recent studies have not demonstrated a clear benefit in reducing anastomotic leaks, making its routine use debatable [22]. Additionally, multimodal prevention of ileus was not included, given the heterogeneous approaches and insufficient high-quality evidence supporting its effectiveness in enhancing recovery after surgery [23]. Routine pelvic drains and nasogastric tubes were discouraged, consistent with both ERAS and ASCRS recommendations, with explicit allowance for selective use under documented clinical indications. These exclusions highlight the need for robust clinical trials to establish the efficacy of such interventions before their integration into standard care protocols.

### Strengths and limitations

Whereas existing international frameworks largely specify institutional or system-level requirements for accreditation and quality assurance, our framework focuses on patient-level interventions that translate such standards into daily clinical practice. By incorporating detailed intraoperative practices and early postoperative milestones, our framework complements organizational metrics with process measures that are directly actionable within care pathways, supporting consistent bedside implementation across both academic and peripheral hospitals. Strengths of this study include a transparent, guideline-anchored methodology, full expert panel participation, and explicit consensus thresholds (≥ 75% agreement) consistent with recommended Delphi practice.

Several limitations merit consideration. First, our framework focuses strongly on clinical process indicators—internal procedures and organizational aspects of care delivery While essential for maintaining consistency and quality, these elements are often not directly visible to patients. To achieve a more integrated and multidimensional understanding of quality, future efforts should combine such process measures with patient-facing components, including communication, education, and shared decision-making [24]. Second, the Delphi design involved a single round with 25 national colorectal surgeons. Although this approach achieved full participation and rapid consensus (≥ 75% agreement), literature indicates that panel composition can shape the resulting key interventions [25]. Third, the literature search was restricted to MEDLINE. While this was judged sufficient to capture major international colorectal guidelines (e.g., NAPRC, ERAS®, ASCRS) and suited the scoping purpose of identifying guideline-based interventions, it may have omitted relevant studies indexed only in Embase or Cochrane.

### Implications and next steps

Integrating the 37 identified key interventions into institutional care pathways is crucial for ensuring consistent, evidence-based implementation of international colorectal surgery standards such as ERAS® and ASCRS guidelines. Embedding these interventions provides a practical foundation for benchmarking and quality improvement, supporting both internal audits and external accreditation efforts. The value of such integration is illustrated by a recent healthcare system-wide collaborative that successfully reduced hospital stays after elective colectomy for cancer through a structured, multi-center pathway approach [26]. Future research should include practice testing (Kotter steps 5–6), international validation, and the incorporation of patient-reported outcomes to complement process indicators. These steps will help transform key interventions into measurable quality indicators and ensure a multidimensional, patient-centered quality framework.

## Conclusion

This study identifies 37 key interventions for rectal cancer surgery, providing a structured framework to strengthen quality of care across the preoperative, intraoperative, and postoperative phases. High expert consensus on critical steps—such as pre- and postoperative multidisciplinary team consultation—underscores their central role in optimizing outcomes. While conceived as candidate quality indicators, many of these elements are best regarded as core clinical or process interventions that form the foundation for future indicator development. Next steps include validation in real-world settings, refinement into measurable indicators where feasible, and integration of patient-centered outcomes. Consistent implementation of these interventions within institutional care pathways will be essential to enhance surgical results and patient satisfaction. 10.7326/M18-0850

## Figures and Tables

**Fig. 2:**
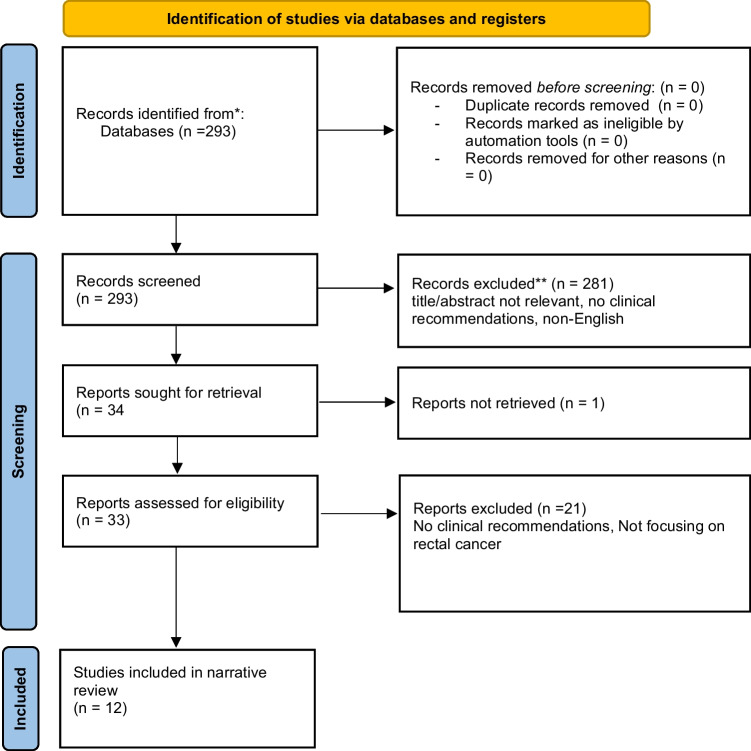
PRISMA 2020 flow diagram summarizing identification, screening, eligibility, and inclusion of literature

## Data Availability

No datasets were generated or analysed during the current study.

## Appendix 1

Figure 2.

## Appendix 2

Table 3.

**Table 3:** Preferred Reporting Items for Systematic reviews and Meta-Analyses extension for Scoping Reviews (PRISMA-ScR) checklist

Section	Item	PRISMA-ScR checklist item	Reported?
**Title**			
Title	1	Identify the report as a scoping review	Yes—Title page
**Abstract**			
Structured summary	2	Provide a structured summary that includes (as applicable): background, objectives, eligibility criteria, sources of evidence, charting methods, results, and conclusions that relate to the review questions and objectives	Yes—Abstract
**Introduction**			
Rationale	3	Describe the rationale for the review in the context of what is already known. Explain why the review questions/objectives lend themselves to a scoping review approach	Yes – Introduction (rationale)
Objectives	4	Provide an explicit statement of the questions and objectives being addressed with reference to their key elements (e.g., population or participants, concepts, and context) or other relevant key elements used to conceptualize the review questions and/or objectives	Yes – Introduction (objectives)
**Methods**			
Protocol and registration	5	Indicate whether a review protocol exists; state if and where it can be accessed (e.g., a Web address); and if available, provide registration information, including the registration number	N/A
Eligibility criteria	6	Specify characteristics of the sources of evidence used as eligibility criteria (e.g., years considered, language, and publication status), and provide a rationale	Yes (methods step 1)
Information sources*	7	Describe all information sources in the search (e.g., databases with dates of coverage and contact with authors to identify additional sources), as well as the date the most recent search was executed	Yes (methods step 2)
Search	8	Present the full electronic search strategy for at least 1 database, including any limits used, such that it could be repeated	Yes (methods step 2)
Selection of sources of evidence†	9	State the process for selecting sources of evidence (i.e., screening and eligibility) included in the scoping review	Yes (methods step 2)
Data charting process‡	10	Describe the methods of charting data from the included sources of evidence (e.g., calibrated forms or forms that have been tested by the team before their use, and whether data charting was done independently or in duplicate) and any processes for obtaining and confirming data from investigators	Yes (methods step 2 & 3)
Data items	11	List and define all variables for which data were sought and any assumptions and simplifications made	Yes (methods step 2 & 3)
Critical appraisal of individual sources of evidence§	12	If done, provide a rationale for conducting a critical appraisal of included sources of evidence; describe the methods used and how this information was used in any data synthesis (if appropriate)	N/A
Synthesis of results	13	Describe the methods of handling and summarizing the data that were charted	Yes (methods step 4)
**Results**			
Selection of sources of evidence	14	Give numbers of sources of evidence screened, assessed for eligibility, and included in the review, with reasons for exclusions at each stage, ideally using a flow diagram	Yes – Results
Characteristics of sources of evidence	15	For each source of evidence, present characteristics for which data were charted and provide the citations	Yes—Results
Critical appraisal within sources of evidence	16	If done, present data on critical appraisal of included sources of evidence (see item 12)	Discussion section/NA
Results of individual sources of evidence	17	For each included source of evidence, present the relevant data that were charted that relate to the review questions and objectives	Yes—Results
Synthesis of results	18	Summarize and/or present the charting results as they relate to the review questions and objectives	Yes—Results
**Discussion**			
Summary of evidence	19	Summarize the main results (including an overview of concepts, themes, and types of evidence available), link to the review questions and objectives, and consider the relevance to key groups	Yes—Discussion
Limitations	20	Discuss the limitations of the scoping review process	Yes—Discussion
Conclusions	21	Provide a general interpretation of the results with respect to the review questions and objectives, as well as potential implications and/or next steps	Yes—Discussion
**Funding**			
Funding	22	Describe sources of funding for the included sources of evidence, as well as sources of funding for the scoping review. Describe the role of the funders of the scoping review	Yes—Acknowledgements

*JBI* Joanna Briggs Institute, *PRISMA-ScR* Preferred Reporting Items for Systematic Reviews and Meta-Analyses Extension for Scoping Reviews

*Where *sources of evidence* (see second footnote) are compiled from, such as bibliographic databases, social media platforms, and Web sites

† A more inclusive/heterogeneous term used to account for the different types of evidence or data sources (e.g., quantitative and/or qualitative research, expert opinion, and policy documents) that may be eligible in a scoping review as opposed to only studies. This is not to be confused with *information sources* (see first footnote)

‡ The frameworks by Arksey and O’Malley (6) and Levac and colleagues (7) and the JBI guidance (4, 5) refer to the process of data extraction in a scoping review as data charting

§ The process of systematically examining research evidence to assess its validity, results, and relevance before using it to inform a decision. This term is used for items 12 and 19 instead of “risk of bias” (which is more applicable to systematic reviews of interventions) to include and acknowledge the various sources of evidence that may be used in a scoping review (e.g., quantitative and/or qualitative research, expert opinion, and policy document)

*From:* Tricco AC, Lillie E, Zarin W, O'Brien KK, Colquhoun H, Levac D, et al. PRISMA Extension for Scoping Reviews (PRISMAScR): Checklist and Explanation. Ann Intern Med. 2018;169:467–473. 10.7326/M18-0850

## Appendix 3

Table 4.

**Table 4:** Mapping of key interventions to possible indicators, evidence level, and data source

Number	Key intervention	Possible indicator	Level of evidence*	Typical data source**
1	Multidisciplinary board preoperatively (preoperative MDT)	% of patients with documented MDT decision before surgery	High (ERAS 2025, ASCRS 2023, NAPRC)	EHR MDT note; cancer registry
2	Patient education and counseling	% with documented counseling on procedure, risks, function	Moderate–high (ERAS, patient-centered care guidelines)	EHR outpatient note
3	Stoma education and positioning	% with stoma site marked and education provided preoperatively	High (ERAS, stoma-care standards)	EHR stoma nurse note
4	Optimization of general condition (smoking cessation, risk assessment, probiotics)	% screened and optimized preoperatively	Moderate/low (ERAS, mixed literature)	Preoperative clinic records
5	Screening nutrition status	% screened with a validated tool (e.g. MUST/NRS)	High (ERAS)	EHR note by Dietitian
6	Oral nutrition supplements	% at-risk patients receiving supplements within one week preoperatively	High (ERAS, meta-analyses)	Dietitians note
7	Screening and treatment of anemia	% screened and corrected (e.g. iron therapy)	High (ERAS, RCT)	Lab results, EHR notes
8	Preoperative MRI	% with high-resolution pelvic MRI	High (ESMO)	Radiology report
9	Intravenous antibiotic prophylaxis	% receiving recommended agent 60 min before incision	High (ERAS, RCT)	Anesthesia report
10	Intraoperative fluid balance	% managed with goal-directed fluid therapy	Moderate–high (ERAS)	Anesthesia report
11	PONV screening (Apfel score)	% documented with Apfel risk score	High (anesthesia guidelines)	Anesthesia report
12	PONV prophylaxis	% receiving prophylaxis per risk score	High (anesthesia guidelines)	Anesthesia report
13	Avoiding sedative medications	% avoiding long-acting sedatives pre/intraoperatively	Moderate–high (ERAS)	Anesthesia report
14	Bower preparation (with/without antibiotics)	% receiving mechanical bowel prep ± oral antibiotics	High (ERAS 2025, ASCRS 2023)	Preop orders; nursing
15	Disinfection of the skin	% with antiseptic skin preparation	High (infection-prevention RCTs)	OR record
16	Minimally invasive surgery	% laparoscopic/robotic resections vs open	High (RCTs/meta-analyses)	OR registry
17	Type of approach: open/laparoscopic/robotic	% cases by approach	High (RCTs)	OR registry
18	Type of reconstruction	% with reconstruction vs permanent ileo/colostomy	Moderate (QOL RCTs)	Operative note
19	Splenic flexure mobilization	% with mobilization documented and AL rate	Moderate (technical standards)	Operative note
20	Visual check anastomosis	% with visual inspection documented and AL rate	Moderate (technical standards)	Operative note
21	Under-water leakage test	% with intraoperative leak test performed and AL rate	Moderate (technical standards)	Operative note
22	Extraction site location	% with documented extraction site and SSI-rate	Moderate (technical standards)	Operative note
23	Use of indocyanine green (ICG)	% with ICG perfusion assessment and AL rate	Moderate (mixed RCT evidence)	Operative note
24	Drain placement	% without routine drain unless indication documented	High (ERAS/ASCRS)	Operative note
25	Stoma placement (temporary vs terminal)	% with type and indication documented	High (stoma-care guidelines)	Operative note
26	High vs low tie mesenteric vein/artery	% with tie level recorded (data collection)	Low (meta-analyses)	Operative note
27	Multimodal pain assessment	% with multimodal analgesia documented	High (ERAS)	Anesthesia report
28	Placement and removal of bladder catheter	Median time to removal: % removed within 48 h	High (ERAS)	EHR note
29	Postoperative mobilization	% mobilized out of bed within 24 h	High (ERAS)	Nursing flowsheet
30	Postoperative nutrition	% started oral fluids/softs within 24 h	High (ERAS)	Nursing record
31	Anastomotic leakage	Rate of clinically significant leak within 90 days	High (quality registry standards)	EHR; surgical registry
32	Re-intervention rate	% requiring re-intervention within 90 days	High (quality registry standards)	EHR; surgical registry
33	90-day readmission/mortality	All-cause readmission or mortality within 90 days	High (international quality indicator)	National registry
34	Postoperative MDT	% discussed at postoperative MDT within 6 weeks	High (oncology/NAPRC standards)	MDT note; cancer registry
35	Quality of TME specimen	% with complete/near complete mesorectal grade	High (pathology standards: NAPRC)	Pathology report
36	Pathology features	% with 12 nodes or more retrieved; % of negative CRM	High (pathology standards: NAPRC)	Pathology report
37	Time to stoma closure	Median time to stoma closure; % closed within 12 months	Moderate–high (observational/QOL data)	EHR; surgical registry

*EHR* electronic health record, *MDT* multidisciplinary team, *OR* operating room, *PONV* postoperative nausea and vomiting, *MBP* mechanical bowel preparation, *OAB* oral antibiotic bowel preparation, *RCT* randomized controlled trial, *CRM* circumferential resection margin, *TME* total mesorectal excision, *QOL* quality of life, *IV* intravenous

*Level of evidence: High = multiple RCTs or international guideline consensus; Moderate = consistent observational/guideline support; Low = expert consensus or limited/conflicting literature

**Typical data source: EHR (clinic, MDT, nursing, operative), OR/anesthesia logs, radiology/lab/pathology reports, pharmacy, hospital administrative or national cancer/surgical registries
